# Development of Assays Using Hexokinase and Phosphoglucomutase Gene Sequences That Distinguish Strains of *Leishmania tropica* from Different Zymodemes and Microsatellite Clusters and Their Application to Palestinian Foci of Cutaneous Leishmaniasis

**DOI:** 10.1371/journal.pntd.0002464

**Published:** 2013-09-26

**Authors:** Kifaya Azmi, Gabriele Schonian, Lionel F. Schnur, Abedelmajeed Nasereddin, Suheir Ereqat, Ziad Abdeen

**Affiliations:** 1 Al-Quds Nutrition and Health Research Institute, Faculty of Medicine, Al-Quds University, Abu-Deis, West Bank, Palestine; 2 Institute of Microbiology and Hygiene, Charité University Medicine Berlin, Berlin, Germany; 3 Kuvin Centre for the Study of Infectious and Tropical Diseases, IMRIC, Hebrew University-Hadassah Medical School, Jerusalem, Israel; US Food and Drug Administration, United States of America

## Abstract

**Background/Objectives:**

Palestinian strains of *L.tropica* characterized by multilocus enzyme electrophoresis (MLEE) fall into two zymodemes, either MON-137 or MON-307.

**Methodology/Principle Findings:**

Assays employing PCR and subsequent RFLP were applied to sequences found in the Hexokinase (HK) gene, an enzyme that *is not* used in MLEE, and the Phosphoglucomutase (PGM) gene, an enzyme that *is* used for MLEE, to see if they would facilitate consigning local strains of *L.tropica* to either zymodeme MON-137 or zymodeme MON-307. Following amplification and subsequent double digestion with the restriction endonucleases *MboI* and *Hae*III, variation in the restriction patterns of the sequence from the HK gene distinguished strains of *L.tropica*, *L.major* and *L.infantum* and also exposed two genotypes (G) among the strains of *L.tropica*: HK-*Lt*G1, associated with strains of *L.tropica* of the zymodemes MON-137 and MON-265, and HK-*Lt*G2, associated with strains of *L.tropica* of the zymodemes MON-307, MON-288, MON-275 and MON-54. Following amplification and subsequent digestion by the restriction endonuclease *MboI*, variation in the sequence from the PGM gene also exposed two genotypes among the strains of *L.tropica:* PGM-G1, associated only with strains of *L.tropica* of the zymodeme MON-137; and PGM-G2, associated with strains of *L.tropica* of the zymodemes MON-265, MON-307, MON-288, MON-275 and MON-54, and, also, with six strains of *L.major*, five of *L.infantum* and one of *L.donovani*. The use of the HK and PGM gene sequences enabled distinction the *L.tropica* strains of the zymodeme MON-137 from those of the zymodeme MON-265. This genotyping system ‘correctly’ identified reference strains of *L.tropica* of known zymodemal affiliation and also from clinical samples, with a level of sensitivity down to <1 fg in the case of the former and to 1 pg of DNA in the case of the latter.

**Conclusions/Significance:**

Both assays proved useful for identifying leishmanial parasites in clinical samples without resource to culture and MLEE.

## Author Summary

The species *L. major*, *L. tropica* and *L. infantum* exist in Palestine and Israel where the first two cause CL and the third usually causes VL although cases of CL without visible signs of VL have been reported from Palestine. This means that diagnosis of locally acquired leishmaniases requires identification of their causative agents for further management of cases. Two molecular biological methods based on sequences from the genes of the enzymes HK and PGM and employing PCRs and consecutive RFLPs were developed and used together to distinguish among strains of the three species and between the two subtypes of *L. tropica* found in Palestinian foci that coincide with zymodemes MON-137 and MON-307. They were applied to, both, isolated parasites grown as promastigotes and to amastigotes in tissue preparations from cases and were able to identify strains and indicate their zymodemal affiliations.

## Introduction

Many methods employing various techniques and targets have been used for the diagnosis of leishmaniases and characterization and identification of their causative agents. Nuclear DNA [1]–[3], miniexon genes [4] kinetoplast DNA [5] and the gp63 gene [6] are among the targets. Multilocus enzyme electrophoresis (MLEE) [7], [8] is the standard accepted method for identifying and distinguishing leishmanial parasites at the species level and is based on variation in the electrophoretic mobility of enzymes isolated from leishmanial organisms. This is performed in a few specialized reference laboratories, which presents some limitations and is expensive and time consuming, requiring large quantities of cultured leishmanial promastigotes. At the end of the procedure, strains are consigned to various zymodemes. Identified Israeli strains of *L. tropica* have fallen into five zymodemes: MON-54., MON-137, MON-265, MON-275 and MON-288, and Palestinian ones into two zymodemes, MON-137 and MON-307. MLEE is not available in Palestinian and Israeli diagnostic clinics so other methods, *i. e.*, nDNA and kDNA analysis [1], [9]–[11] and excreted factor (EF) serotyping [12] have been used. Though these tend to parallel characterization by MLEE, they cannot be linked directly to the genetics underlying the enzymes examined by MLEE.

Here, sequences from the genes for Hexokinase (HK) and Phosphoglucomutase (PGM) have been tested as targets for methods incorporating, consecutively, a polymerase chain reaction (PCR) and restriction fragment length polymorphism (RFLP) to identify strains of the three Old World leishmanial species *L. major*, *L. tropica* and *L. infantum* and attempt to differentiate among strains of *L. tropica* and indicate to which zymodemes they probably belong, and also apply the two techniques to the diagnosis of leishmaniases. Genotyping results derived through the PCR RFLP system were firstly validated using reference strains and then applied to clinical samples from cases of CL.

## Materials and Methods

### Isolation of parasites, clinical samples and DNA extraction

The 95 strains of *L. tropica* used were from different geographical areas (Table 1). Thirteen of which represented five different zymodemes [8], [13], [14]. In addition, Drs. Christophe Ravel and Gert de Auwera, Université Montpellier 1, Centre Hospitalier Universitaire de Montpellier, Laboratoire de Parasitologie and Centre National de Reference des *Leishmania*, Montpellier, France, provided DNA samples from 17 strains. Forty-five strains of *L. tropica* of unknown zymodemal affiliation were analyzed by the HK and PGM PCR RFLP assays developed at Al-Quds University. Ten of the Palestinian strains of *L. tropica* analysed had been characterized by MLEE (Table 1) and [14]. Six strains of *L. major*, (MHOM/IL/1989/P241, MHOM/PS/2008/358JnQF36, MHOM/PS/2008/359JnM22, MHOM/SU/1973/5ASKH, MHOM/IL/1980/Friedlin, MHOM/PS/1967/JerichoII), five of *L. infantum* (MHOM/TN/1980/IPT1, MHOM/TR/1994/EP3, MCAN/IL/2011/LRC-L1500, MCAN/PS/2011/dogK6, MHOM/PS/2004/LQU217 and one of *L. donovani* (MHOM/SD/????/Khartoum), were used as out groups.

**Table 1:** The HK and PGM genotypes of the strains of *Leishmania tropica* studied, listed by their WHO codes, in which M stands for mammal, HOM *Homo sapiens*, PRO the hyrax genus *Procavia*, I insect, SER the sand fly species *Phlebotomus sergenti*, ARA the sand fly species *Ph. arabicus*, ROS the sand fly species *Phlebotomus rossi*, EG Egypt, IL Israel, PS Palestine, KE Kenya, YE Yemen, NA Namibia, IQ Iraq, TR Turkey, IR Iran, AZ Azerbaijan, SY Syria, SU ex-Soviet Union, AF Afghanistan, MA Morocco, JO Jordan, IN India.

PGM	HK	Zymodeme		WHO strains codes		Total
			MHOM/EG/1990/LPN65	MHOM/IL/1998/LRC-L747	MHOM/IL/1997/P963	
			ISER/IL/1998/LRC-L758	ISER/IL/2004/LRC-L1155	MHOM/IL/2006/LRC-L1186	**18**
		**MON-137**	MHOM/IL/1996/P837	MHOM/PS/2002/87JnYM	MHOM/PS/2002/89JnF	
			MHOM/PS/2002/5JnYF5	MHOM/PS/2002/79JnSF20	MHOM/PS/2002/64JnSF4	
			MHOM/PS/2002/34JnSF4	MHOM/PS/2002/31JnTM17		
			MHOM/PS/2010/LRUJ-1703	MHOM/PS/2002/35JnYF45	MHOM/PS/2000/GOKS17	
			MHOM/IL/2010/LRC-L1458	MHOM/PS/2009/384JnFS	MHOM/PS/2008/340JnTyF7	
			MHOM/PS/2008/336JnSM71	MHOM/PS/2003/186JnBM12	MHOM/PS/2003/151JnTyF32	
			MHOM/PS/2008/332JnYM37	MHOM/PS/2008/344JnSF53	MHOM/PS/2003/167JnYF14	
			MHOM/PS/2003/152JnTyF32	MHOM/PS/2011/462JnYM41	MHOM/PS/2011/455JnYM19	
**PGM-G1**	**HK-** ***Lt*** **G1**	**ND**	MHOM/PS/2002/50JnYM20	MHOM/PS/2011/456JnM16	MHOM/PS/2003/185JnSeM27	**45**
			MHOM/PS/2011/452JnSF48	MHOM/PS/2003/178JnYM79	MHOM/PS/2011/454JnAF6	
			MHOM/PS/2011/457JnQM18	MHOM/PS/2011/461JnSF14	MHOM/PS/2010/391Jn	
			MHOM/PS/2003/184JnY01	MHOM/PS/2008/335JnYM59	MHOM/PS/2001/ISL593	
			MHOM/PS/2011/456JnM16	MHOM/PS/2011/467Jn	MHOM/PS/2010/409JnF	
			MHOM/PS/2008/tareq	MHOM/PS/2009/389JnM	MHOM/PS/2011/LRUJ-1781	
			MHOM/PS/2011/LRUJ-1779	MHOM/PS/2010/LRC-L1449	MHOM/PS/2010/395Jn	
			MHOM/PS/2003/ISLAH721	MHOM/PS/2010/411JnM55	MHOM/PS/2010/423JnM36	
			MHOM/PS/2010/LRC-L1448	MHOM/PS/2001/ISLAH590	MHOM/PS/2010/410Jn20	
			MHOM/PS/2010/416JnM39	MHOM/PS/2010/426JnM	MHOM/PS/2010/413JnF	
		**MON-265**	MHOM/IL/2002/LRC-L863	IARA/IL/2000/LRC-L810		**5**
		**ND**	ISER/IL/2002/LRC-L909	IARA/IL/2002/LRC-L910	ISER/IL/2002/LRC-L907	
**PGM-G2**	**HK-** ***Lt*** **G1**	**MON-119**	MHOM/KE/1991/EB135			**1**
		**MON-71**	MHOM/YE/1986/LEM1000			**1**
		**ND**	IROS/NA/1980/HD3			**1**
			MHOM/NA/1984/K1			**1**
		**MON-6**	MHOM/IQ/1966/BRAY-L75			**1**
		**MON-53**	MHOM/TR/1999/EP32			**1**
		**MON-39**	MHOM/IR/1995/5043			**1**
		**MON-61**	MHOM/IR/2000/LEM4036			**1**
		**MON-75**	MHOM/AZ/1958/OD			**1**
		**MON-76**	MHOM/SY/1995/LSL25			**1**
		**MON-55**	MHOM/SU/1966/III			**1**
		**MON-60**	MHOM/SU/1974/SAF-K27			**1**
		**MON-58**	MHOM/AF/1988/KK26			**1**
		**MON-102**	MHOM/MA/1995/UERL9			**1**
		**MON-109**	MHOM/MA/1989/LEM1781			**1**
**PGM-G2**	**HK-** ***Lt*** **G2**	**MON-112**	MHOM/MA/1992/LPN79			**1**
		**MON-264**	MHOM/MA/1995/LEM3015			**1**
		**MON-275**	MHOM/IL/1990/P283			**1**
		**MON-288**	MHOM/IL/1959/LRC-L22			**1**
		**MON-54**	MHOM/IL/1980/Singer ( = LRC-L303)		1	
			MOHM/PS/2002/18JnMF4			
		**MON-307**	MHOM/PS/2002/20JnYM3			**3**
			MHOM/PS/2002/52JnYM18			
		**ND**	MHOM/JO/2009/LRUJ-1550			**2**
			MHOM/JO/2009/LRUJ-1564			
		**LON-17**	MHOM/KE/1981/NLB030B			**1**
		**LON-12**	MHOM/IN/1979/DD7			**1**
**Total**						**95**

Sixty-one cases of CL diagnosed on clinical grounds only and registered as such by the Palestinian Ministry of Health (PMOH) were later confirmed, using their tissue aspirates that had been spotted onto filter papers. This included identification of the parasites. Genomic DNA was extracted from pellets of promastigotes and from amastigotes in skin tissue aspirates collected on filter papers. A high purification template PCR kit (Roche Diagnostics GmbH, Mannheim, Germany) was used and the DNA was eluted in 40 µl Tris EDTA buffer (10 mM Tris, 1 mM EDTA, pH 8.0). The use of patient samples was approved by the Research Ethics Committee of Al- Quds University, Jerusalem. All the reference strains (Table 1) and only the positive clinical samples that had been confirmed previously and had had their parasites identified by an ITS1PCR and consecutive RFLP analysis [1] were analyzed here. To evaluate the possibility of employing the HK and PGM PCR RFLP assays established here without isolating the parasites, 61 tissue aspirates from positive cases caused by *L. tropica* spotted onto filter paper were assessed.

### Selection of oligonucleotides

A combined analytical approach was used to differentiate between the species *L. tropica*, *L. major* and, *L. infantum* and, in particular, to separate Palestinian strains of *L. tropica* of different zymodemal affiliation. This was done by carrying out an HK PCR RFLP assay and a PGM PCR RFLP assay.

To design the primers for the HK PCR, all the available data on leishmanial sequences of the Hexokinase gene were aligned (accession numbers, AY632240.1, AY632239.1, AY635845.1, XM 001682906, XM 001682905.1, XM 001564691.1, XM 001465299.1, XM 001465298.1, AM502239, AM494958.1, AY676310.1, AY632241.1, AY659996.1), using the multalin, National Center for Biotechnology Information (NCBI) (http://www.ncbi.nlm.nih.gov/). Primers were designed with Primer 3 software (http://frodo.wi.mit.edu), which enabled identification of specific sequential elements for the design of appropriate primers that increase specificity and would be suitable for RFLP. This was owing to the need to differentiate *L. tropica* from *L. major* and these from *L. infantum*/*L. donovani* and to discriminate between zymodemal affiliations of the strains, especially of the two known zymodemal variants of Palestinian strains of *L. tropica*. The primer sequences were: HKF (Forward), 5′-CCAACGCCTGCTACTTTGAG-3′, and HKR (Reverse), 5′-CTTCTCTTGGCGCTGGTTCT-3′.

GenBank listed sequences of PGM for *L. major* and *L. infantum* but none had been published for *L. tropica*. In this study, the sequences of PGM from two strains of *L. tropica* belonging to the zymodeme MON-307, MHOM/PS/2002/18JnF4 and MHOM/PS/2002/20JnM3, and three belonging to the zymodeme MON-137, MHOM/PS/2002/87JnM, MHOM/PS/2002/64JnF4 and MHOM/IL/1990/P283, were revealed by amplifying and sequencing the target using PGM4F:5′-GAGGTGACAACGACAGCGTA-3′, and PGM172R:5′- GGCCAGTCAGAGATTCCATC-3′ and the internal primer PGM1124R:5′- AAGAACTTCCACCCCGTAGG-3′ to obtain the forward and reverse sequences for the entire gene. This generated a 1700 bp amplicon. These sequences were used as references for this study. Samples were purified using a PCR product purification kit (Roche Diagnostics GmbH, Mannheim, Germany). The amplicons were aligned against the sequences of *L. major* and *L. infantum* and the nucleotide differences among strains of *L. tropica* were exposed, using the CLUSTAL program (.http://multalin.toulouse.inra.fr/multalin/) The primers PGMLtF:5′- TCCGTGAGAAGGACGGTATC-3′ and PGMLtR:5′-AGGGTCCGTGTAGCTGAAGT-3′ were used in a PCR RFLP assay to reveal the sequence that differentiates strains of *L. tropica* of the zymodeme MON-137 from those of the other zymodemes explored here.

### PCR amplification conditions for HK and PGM sequences

HK and PGM PCR reactions were done in a volume of 25 µl that contained 1 µl of DNA template and PCR-Ready Supreme mix (Syntezza Bioscience, Jerusalem, Israel) in a Gene Amp PCR-system 9700 thermal cycler (Applied Biosystems, CA, USA), using the following conditions: initial denaturation for 5 min. at 98°C, followed by 38 cycles at 94°C for 45 s, 55°C for 45 s, and 72°C for 30 s and final elongation at 72°C for 7 min. For each reaction, DNA from strains of *L. major* and *L. infantum*, and strains of *L. tropica* belonging to the various zymodemes mentioned above were used as positive controls and distilled water was used as a negative control. Ten µl of each PCR product were run in 2.5% agarose gels. For all the species of *Leishmania*, the amplified products of the HK sequence were 197 bp and those of the PGM sequence were 278 bp.

### Selection of the HK and PGM PCR restriction endonucleases

To select appropriate endonucleases for distinguishing between strains of *L. infantum*, *L. major*, and *L. tropica*, reference sequences of HK from GenBank were used. The NEBcutter V2.0 program available at the website http://tools.neb.com/NEBcutter2/index.php was able to differentiate these species. Theoretically, a double digestion of the PCR product with the endonucleases *HaeIII* and *MboI* should give three different digestion patterns, each corresponding to the three species and, as digestion with just *MboI* proved insufficient to separate *L. tropica* and *L. infantum*, double digestion with *MboI* and *HaeIII* was carried out, in the same tube at the same time.

The PGM gene sequences of all the strains of *L. tropica* were mapped for restriction site polymorphisms. The restriction enzyme *MboI* was selected to produce fragment sizes able to separate strains of *L. tropica* of the zymodeme MON-137 from all the other types of strain examined, which included strains of *L. tropica* that were not of the zymodeme MON-137 and strains of *L. major* and *L. infantum*.

For, both, the HK PCR RFLP and the PGM PCR RFLP, 15 µl of PCR product were incubated with 10 U endonuclease (MBI Fermentas), i. e., *Mbo*I and *Hae*III, together in the same tube at the same time in the former and, only *Mbo*I for the latter, for 2 hr at 37°C, according to the manufacturer's instructions. Digestion products were electrophoresed in a 3% agarose gel (FMC BioProducts, Rockland, ME) in TAE buffer. Digestion patterns were stained with ethidium bromide and visualized by UV light. A DNA 50 bp molecular weight ladder (Promega, Madison, WI) was added to gels to indicate the molecular size of the digestion fragments.

### Sensitivity and specificity of the HK PCR and PGM PCR

To determine the minimal amount of leishmanial DNA needed for amplification of the HK and PGM gene sequences by their respective PCRs for their RFLP analyses, each of a series of tenfold dilutions of leishmanial DNA extracted from the promastigotes of the strains LRC-L890 and -L758 was amplified. Concentrations of DNA were measured by a Nanodrop ND-1000 UV-Vis spectrophotometer (Thermo Fisher Scientific Inc, Waltham MA, USA). A search for the similarity in genetic sequences in GenBank revealed 100% specificity of the primers used for strains of *Leishmania*.

### Sequencing of Hexokinase

**Figure 1:**
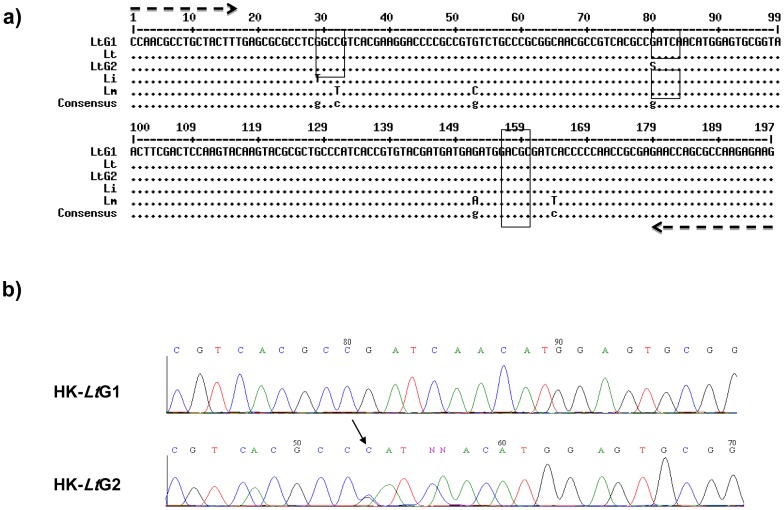
a) Alignment of the HK nucleotide sequences of the strains of *Leishmania tropica*, *L. major* and *L. infantum*: cleavage sites of endonucleases MboI and HaeIII are ‘boxed’ in the HK sequence of the strains of *L. tropica*: HK-*Lt*G1 = *L. tropica* genotype 1 derived from strain ISER/IL/1998/LRC-L758; Lt = *L. tropica* DNA sequence, accession number = AY635845.1; HK-*Lt*G2 = *L. tropica* genotype 2 derived from strain MHOM/PS/2002/52JnYM18 ( = LRC-L890) Li = *L. infantum*, accession number = AY632240.1; Lm = *L. major*, accession number = FR796417.1. Arrows indicate positions of HK forward and reverse primers. b) Electropherograms of the sequences bestowing the *L. tropica* genotypes 1 and 2, HK-*Lt*G1 and HK-*Lt*G2, respectively. The pinpointed (↓) C at the bp 80 shows a double peak for (G–C) in the HK-*Lt*G2 electropherogram and should have been denoted by an S instead of the C. NN in the HK-*Lt*G2 electropherogram indicates (C) and (A) nucleotide, respectively.

The PCR amplification products from the strains *L. tropica* ISER/IL/1998/LRC-L758 and *L. tropica* MHOM/PS/2002/LRC-L890, representing the zymodemes MON-137 and MON-307, respectively, were purified with a PCR product purification kit (Roche Diagnostics GmbH, Mannheim, Germany) and sequenced (Applied biosystem, genetic analyzer, Foster City, CA). The sequences were processed and aligned, using the CLUSTAL software package, and nucleotide differences were detected with the programs BioEdit and Clustal X (Fig. 1).

### Ethical statement

A total of sixty-one tissue aspirates spotted onto filter papers were collected from CL patients. All tested samples were anonymized. The study protocol was approved by the Research Ethics Committee of Al-Quds University.

### Accession numbers

AY632240.1, AY632239.1, AY635845.1, XM 001682906, XM 001682905.1, XM 001564691.1, XM 001465299.1, XM 001465298.1, AM502239, AM494958.1, AY676310.1, AY632241.1, AY659996.1, HQ141924, HQ141925, HQ141926, HQ141927, HQ141928, AY635845.1, AY632240.1, FR796417.1.

## Results

### PCR RFLP of the HK gene

**Figure 2:**
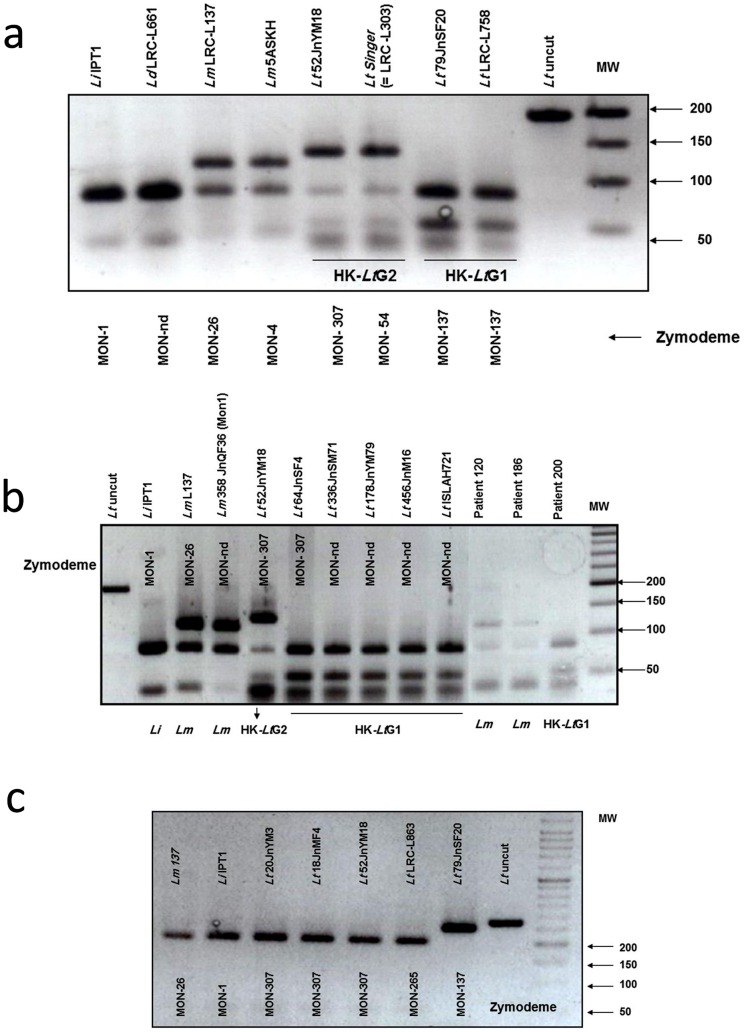
Agarose gel electrophoresis of : a) the PCR products from the Hexokinase (HK) genes of leishmanial reference strains after their double digestion with the endonucleases *MboI* and *HaeIII*, nd = not done; b) of PCR products from the HK genes of the leishmanial references and local Israeli and Palestinian strains, and Palestinian clinical samples after their double digestion with the endonucleases *MboI* and *HaeIII*. Uncut PCR product from *L. tropica* MHOM/PS/18JnMF4 ( = LRC-L891) was used as a control and molecular sizes were compared with a 50 bp molecular weight ladder (MW), nd = not done; c) of the PCR products from the Phosphoglucomutase (PGM) genes of the leishmanial reference strain *L. tropica* LRC-L863 and local Palestinian strains after single digestion with the endonuclease *MboI*. Only the heavier differentiating band is shown. Uncut PCR product from *L. tropica* MHOM/PS/02/79JnF20 ( = LRC-L885) was used as a control and molecular sizes were compared with a 50 bp molecular weight ladder (MW).

RFLP analysis of the 197 bp amplicon from the HK gene of the reference strains of *L. major*, *L. infantum, L. donovani* and the two zymodemal types of *L. tropica* gave their expected patterns, and different restriction fragment patterns were generated for the three different species and between the two zymodemal types of *L. tropica* (Figs. 2a and 2b).

The digested amplicons of the strains of *L. tropica* gave two different restriction patterns: those, whose profiles displayed four bands of 81, 49, 37, 30 bp, of which the latter two appear as a single broad band, that were assigned to the genotype HK-*Lt*G1 and, co-incidentally, belonged to either zymodeme MON-137 or zymodeme MON-265; and those, whose profile displayed the same four bands of 81, 49, 37, 30 bp plus an extra one of approximately 130 bp that were assigned to the genotype HK-*Lt*G2 and, co-incidentally, belonged to the other zymodemes mentioned above (Fig. 2a, Table 1). This was confirmed by sequence analysis of the PCR products of the two strains of *L. tropica*, ISER/IL/1998/LRC-L758 and MHOM/PS/2002/52JnM18 ( = LRC-L890) representing the genotypes HK-*Lt*G1 and HK-*Lt*G2, respectively, where the latter showed a C/G alteration at position 80 compared with the former (Figs. 1a and 1b). This HK PCR RFLP was done on more than one sample and reproducible results were obtained, indicating that strains of *L. tropica* contain both alleles, C and G. When it is G, two bands appear, when C, digestion does not occur and no bands are seen. When both alleles, C and G, are present as indicated by the conventional S, three bands are seen.

### PCR RFLP of the PGM gene and its sequencing

The sequences of the PGM of the five strains of *L. tropica*, *i. e.*, the strains MHOM/PS/2002/87JnM and MHOM/PS/2002/64JnF4 of the zymodeme MON-137, and the strains MHOM/PS/2002/20JnM3 and MHOM/PS/2002/18JnF4 of the zymodeme MON-307, and the strain MHOM/IL/1990/P283 of the zymodeme MON-275 are in Genbank under the accession numbers HQ141924 to HQ141928. When their sequences were aligned, those of the first two strains listed above were of the genotype PGM-G1 and those of the other three strains were of the genotype PGM-G2, and there were twelve heterozygous loci with multiple heterozygous sites between the two genotypes (Data not shown).

The six strains of *L. major*, five of *L. infantum* and five of *L. tropica* all yielded an amplicon of 278 bp. Digestion of the amplicons with just *MboI* and subsequent comparison of the RFLP patterns showed no significant difference between *L. major* and *L. infantum* and only two restriction bands of 204 and 50 bp, for *L. major*, and 204 and 56 bp, for *L. infantum*, were seen. A third band of about 24 bp, for *L. major* and of about 18 bp for *L. infantum*, also exists and though not clearly seen to be different in the gel was shown to be so by the Neb Cutter program. This consigned the strains of *L. major* and *L. infantum* to the genotypes PGM-G2. The strains of *L. tropica* MHOM/PS/2002/87JnM and MHOM/PS/2002/64JnF4, aligned with the zymodeme MON-137, exhibited fragments of 254 bp and 24 bp and, thus, were genotype PGM-G1. The strains of *L. tropica* of MHOM/PS/2002/20JnM3, and MHOM/PS/2002/18JnF4, aligned with the other zymodemes mentioned in this study to which strains of *L. tropica* belonged, exhibited fragments of 204 bp and 56 bp and, thus, were genotype PGM-G2, a characteristic that they shared with strains of *L. major* and *L. infantum*. (Table 1 and Fig. 2c).

### Sensitivity and specificity of the HK PCR and PGM PCR

**Figure 3:**
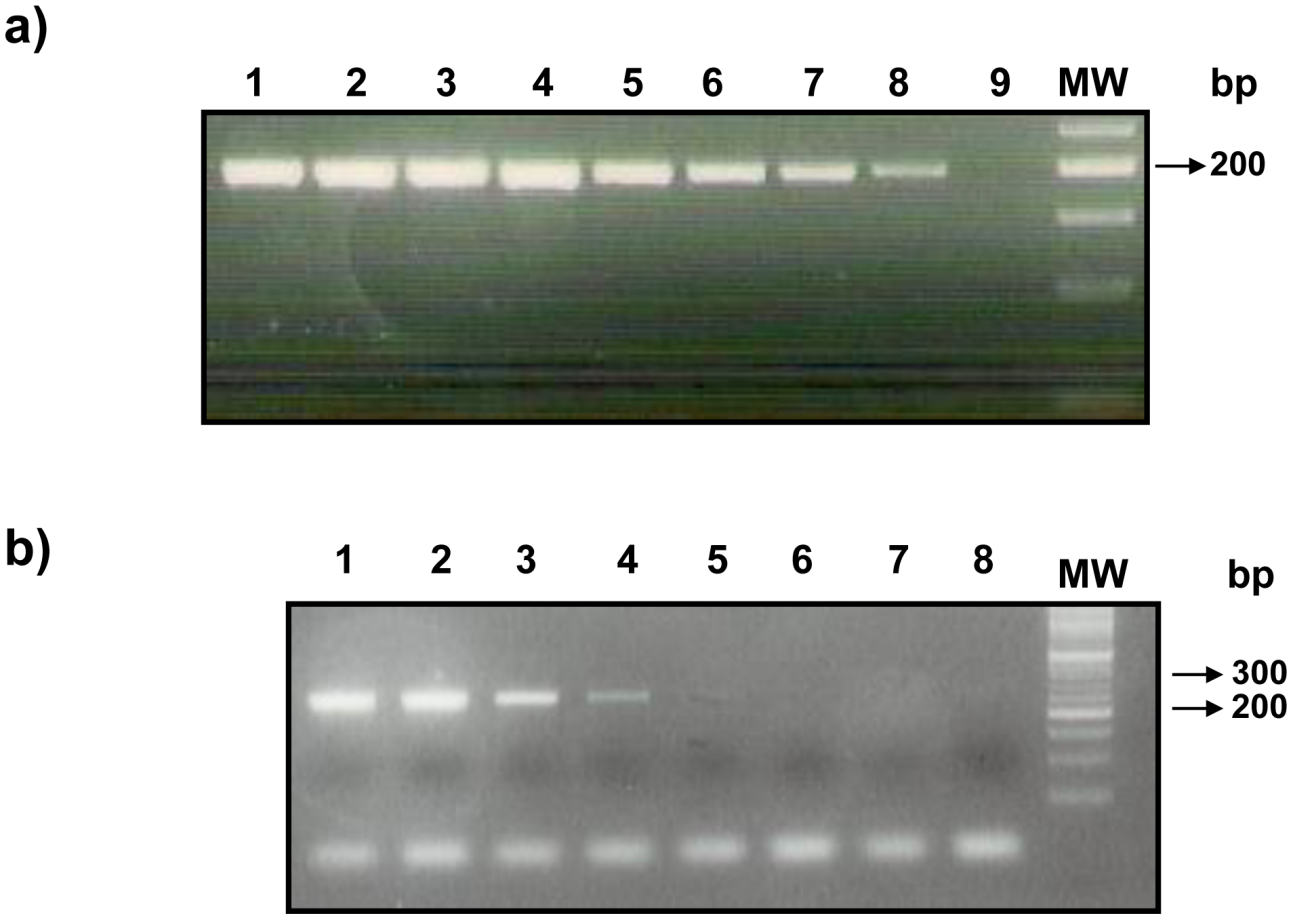
a) Analytical sensitivity of the PCR amplification of the Hexokinase (HK) gene (product size 197 bp). Lanes 1–8 were 10-fold dilutions of leishmanial genomic DNA from 10 ng to 1 fg per reaction. Lane 9 was the negative control and molecular sizes were compared with a 50 bp molecular weight ladder (MW). b) Analytical sensitivity of the PCR amplification of the Phosphoglucomutase (PGM) gene (product size 278 bp). Lane 1–8 were 10-fold dilutions of leishmanial genomic DNA from 1 ng to 1 fg per reaction. Molecular sizes were compared with a 50 bp molecular weight ladder (MW).

For the PGM sequence of the strains LRC-L890 and -L758, a 278 bp PCR product was clearly detected at a dilution of the genomic DNA that contained an estimated 1 pg of DNA, which is considered to be equivalent to 10 leishmanial parasites. The minimal amount of amplified DNA detectable by the HK PCR was less than 1 fg of DNA, which is considered to be equivalent to 0.01 leishmanial parasites [15] (Figs. 3a and b). Other cutaneous infections that could be confused clinically with CL are bacterial and fungal infections, for example, staphylococcal and streptococcal infections. However, regarding the clinical specificity and utility of this leishmanial parasite-specific PCR-RFLP diagnostic assay, a BLAST search gave perfect matches with 100% query coverage identity for the HK sequence of leishmanial species amplified with the primers used in the HK PCR. It also gave 100% coverage identity with the registered sequence XM 001682945.1 from *L. major* and the three registered sequences XM 001564729.1, XM 001465338.1, AM 50223.91 from *L. infantum*. The BLAST search also gave perfect matches with 100% query coverage identity for the PGM sequence of leishmanial species amplified with the primers used in the PGM PCR. It also gave 100% coverage identity with the registered sequence from *L. donovani* FR799608.1, from *L. major* FR796455.1 and *L. major* XM001682945.1, from *L. infantum* FR796455.1, and from *L. mexicana* FR799574.1. Sequences from *Trypanosoma cruzi*, African trypanosomes, *Crithidia luciliae*, *Crithidia fasiculata*, and *Leptomonas collosoma* were not found in the GenBank Megablast and could not be compared. No amplification products were observed when either the DNA of *Trypanosoma cruzi* or human DNA was used as templates in this PCR approach (data not shown).

### RFLP analysis of the HK and PGM gene sequences from local and foreign strains of *Leishmania*

The restriction profiles of 73 out of 99 DNA samples amplified by the HK PCR and doubly digested with *MboI* and *HaeIII* were identical to that of the reference strain of *L. tropica* ISER/IL/1998/LRC-L758, representing the zymodeme MON-137, and were genotype HK-*Lt*G1; 26 were identical to that of the reference strain of *L. tropica* MHOM/PS/2002/52JnM18 ( = LRC-L890), representing the zymodeme MON-307, and were genotype HK-*Lt*G2. Ten of the 55 Palestinian strains of *L. tropica* had been analyzed by MLEE, of which seven (MHOM/PS/2002/31JnTM17, MHOM/PS/2002/5JnYF5, MHOM/PS/2002/34JnSF4, MHOM/PS/2002/79JnSF20, MHOM/PS/2002/87JnYM, MHOM/PS/2002/64JnSF4, MHOM/PS/2002/89JnF) belonged to the zymodeme MON-137 and three (MHOM/PS/2002/52JnYM18, MHOM/PS/2002/20JnYM3, MHOM/PS/2002/18JnMF4) to the zymodeme MON-307. Amplification of the PGM sequence from the former seven and its digestion with *MboI* alone gave a profile with a product of 254 bp and that from the latter three gave a profile with a product of 204 bp. This was congruent with results from the HK PCR RFLP, where the seven strains of the zymodeme MON-137 and, also, strains of *L. tropica* from the zymodeme MON-265 gave the restriction profile displayed by the reference strain of *L. tropica* of the genotype HK-*Lt*G1 and the three strains from the zymodeme MON-307 gave the restriction profile displayed by the reference strain of *L. tropica* of the genotype HK-*Lt*G2 The other 41 strains of *L. tropica* analyzed gave the restriction profile displayed by the reference strain *L. tropica* ISER/IL/1998/LRC-L758, representing the zymodeme MON-137, assigning them to the genotype HK-*Lt*G1 (Table 1).

The PGM PCR RFLP profiles of the 11 Israeli strains of *L. tropica*, whose zymodemal affiliation was known and belonged one of the zymodemes MON-137, MON-265, MON-288, MON-275 and MON-54, that were genotyped blindly, separated into two genetic groups: those that had PGM PCR RFLP profiles indicating the genotype PGM-G1 and were like strains shown to be strains of the zymodeme MON-137; and those that had PGM PCR RFLP profiles indicating the genotype PGM-G2 and were like strains shown to be strains of the zymodemes MON-265, MON-288, MON-275 MON-54 and MON-307 (Fig. 2c). As mentioned above, all the strains analyzed by MLEE and shown to be of the zymodeme MON-137 and the two shown to be of the zymodeme MON-265 produced HK PCR RFLP profiles of the genotype HK-*Lt*G1. However, the strains of the zymodemes MON-288, MON-275, MON-54 produced HK PCR RFLP profiles of the genotype HK-*Lt*G2.

Interestingly, 19 strains of *L. tropica* of known zymodemal type and four whose zymodemal type was not known, all from geographical regions other than Palestine and Israel, displayed PGM PCR RFLP profiles, indicating they were genotype PGM-G2 and HK PCR RFLP profiles, indicating they were genotype HK-*Lt*G2 (Table 1).Whereas, the strains belonging to the zymodemes MON-119 and 71 from Kenya and Yemen, respectively, and the two Namibian strains of unknown zymodemal type, were genotype PGM-G2 but genotype HK-*Lt*G1 (Table 1).

### Application of the HK and PGM PCR RFLP assays to clinical samples

Of the 61 positive aspirates amplified by the HK and PGM PCRs, 56 gave a product of 197 bp and 43 gave a product of 278 bp, respectively. All the PCR positive samples were subjected to HK RFLP analysis of their amplicons in comparison to the reference strains and consigned to their species. Forty-six of the 56 samples were *L. tropica*. The restriction profile and corresponding genotype, HK-*Lt*G1, were like those of the reference strains of the zymodemes MON-137 and MON-265. Thirty-eight of the 46 samples that were positive by the HK PCR were also identified by PGM RFLP analysis and were genotype PGM-G1 like the reference strains of the zymodeme MON-137 (data not shown).

## Discussion

Identification of leishmanial species is an integral part of the diagnosis of leishmaniases. Many methods have been used for this and together they have revealed considerable micro-heterogeneity within each different species. For example, strains of *L. tropica* show considerable variation in their microsatellite profiles [16] and in their enzyme profiles that have consigned them to 35 different zymodemes to date [7], [14], [17], [18]. The strains of *L. tropica* causing CL in Palestine display two types of enzyme profile, affirming the existence of two zymodemes, MON-137 and MON-307 [14], that coincide with genetic clusters separated by multilocus microsatellite typing (MLMT) [14]. Strains of the zymodeme MON-137 have also been isolated in Israel as have strains of the four zymodemes MON-288, MON-275, MON-265 and MON-54. MLEE showed that most of the Palestinian strains of *L. tropica* considered here belonged to the zymodeme MON-137, strains of which in addition to occurring in Israel also occur in Jordan and Egypt [7], [11], [19], [20]. Strains of *L. tropica* of the zymodeme MON-307 have not been isolated in these countries or any other countries where leishmaniases occur.

Of the two enzymes used here in PCR RFLP analyses, one, PGM, is included among the 15 enzymes used to generate enzyme profiles according to the system applied at the ‘Centre National de référence des *Leishmania*’ in Montpellier, the other, HK, is not. The ability to distinguish the species, *L. major, L. tropica and L. infantum* from one another by employing one DNA sequence from the gene for HK compared favourably with the separation of these species by cellulose acetate enzyme analysis (CAE) when using only the HK enzyme where Kreutzer [21],[22] did not manage to separate these three species. However, Mebrahtu [23], applying the same CAE system, did manage to distinguish the species, *L. major, L. tropica and L. infantum* from one another using the HK enzyme. Also, employing this one DNA sequence from the gene for HK enabled the separation of strains of *L. tropica* into two genotypes associated with different groups of zymodemes (Table 1).

RFLP analysis before and, even more so, after the introduction of the polymerase chain reaction has proved very useful in identifying pathogens like leishmanial parasites as isolates from clinical samples, vectors and animal hosts and *in situ* in infected tissues [24]. The HK PCR RFLP developed here was, in addition, able to indicate genetic sub-types of the species *L. tropica* that appear to be directly related to zymodemal sub-types [1], which might be of epidemiological significance. However, for routine diagnostic use, it is difficult to achieve the required level of sensitivity and discrimination in a single step. Umasankar et al. [25], have shown that the HK gene displays sufficient polymorphism to be able to use sequencing of hexokinase loci to distinguish the species, *L. major, L. tropica and L. infantum*, which are the species circulating in Palestine. A sequence from the HK gene was selected here, which, in addition, also separated strains of *L. tropica* into two previously exposed genotypes: HK-LtG1, associated with the strains of the zymodemes MON-137 and MON-265; and HK-LtG2, associated with the strains of the zymodemes MON-307, MON-288, MON-275 and MON-54 [14].

This method easily distinguished between the Palestinian strains of the zymodemes MON-137 and MON-307, the only two zymodemes shown to occur in the Jenin District of Palestine [26] and one wonders if all the Palestinian strains examined here that were either genotype HK-*Lt*G1 or genotype HK-*Lt*G2 would prove to be, respectively, strains of the zymodemes MON-137 (or, possibly, MON-265, not found in Palestine so far) and MON-307 (or, possibly, MON-288, MON-275, MON-54, also not found in Palestine so far) if their enzyme profiles were determined.

At this time, the clinical and epidemiological relevance of being able to distinguish between strains of *L. tropica* belonging to zymodemes MON 137 and MON 265 is uncertain but could prove significant with continued epidemiological and chemotherapeutic studies. Presently, it is of biological interest and useful for population genetics. There seems to be no precise correlation between clinical form and species of *Leishmania*. A single species and even strains within a single zymodeme can cause lesions of different style and the same type of lesion can be produced by strains of different leishmanial species [7]. The lack of correlation between clinical presentation and enzyme polymorphism of *L. infantum* has been noted Pratlong [27]. Also, at this time, chemotherapy would be the same irrespective of the leishmanial species. Regarding epidemiology, the sand fly vector(s) transmitting the two sub-types of the Palestinian strains of *L. tropica* described here have not been identified. It would be well to know if they are transmitted by the same vector or each sub-type of *L. tropica* has a different species of vector. For example, in Israel, *Phlebotomus* (*Adlerius*) *arabicus* was shown to be sand fly vector of *L. tropica* of the zymodeme MON-265 in a focus just north of the Sea of Galilee [28]–[30] whereas *Phlebotomus* (*Paraphlebotomus*) *sergenti* was shown to be sand fly vector of *L. tropica* of the zymodeme MON-137 [11].

**Figure 4:**
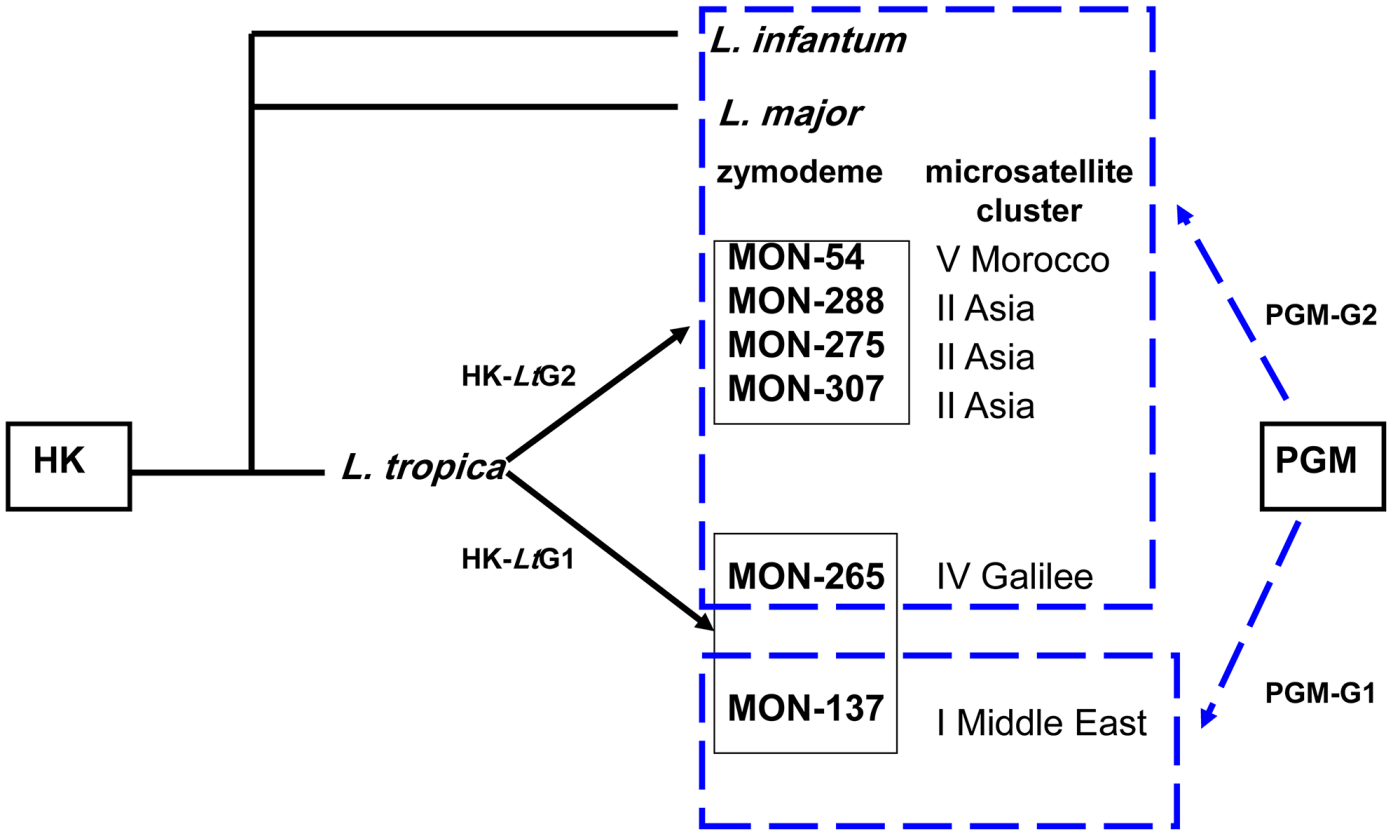
Scheme of the interrelationship between the genotypes derived from the PCR RFLP profiles of the chosen hexokinase gene sequence after double digestion of the PCR product with *MboI* and *HaeIII* and the phosphoglucomutase gene sequence after single digestion of the PCR product with just *MboI*, and their correlation to the zymodemes and microsatellite clusters encompassing Palestinian and Israeli strains of *L. tropica*: genotype HK-*Lt*G1 = Hexokinase *L. tropica* group 1, genotype HK-*Lt*G2 = Hexokinase *L. tropica* group 2, genotype PGM-G1 = Phosphoglucomutase group 1, genotype PGM-G2 = Phosphoglucomutase group 2. Zymodemal designations were taken from, Rioux [8] and Pratlong [7], and microsatellite clusters from Schwenkenbecher [16].

The RFLP profiles of the HK sequences of these two zymodemal types of strain of *L. tropica*, generated after digesting their PCR amplicons were congruent with the DNA sequencing (Fig. 2). The PGM PCR RFLP assay was not suitable for distinguishing the leishmanial species mentioned above, but it did separate strains identified as strains of *L. tropica* belonging to the zymodeme MON-137 from all the strains of *L. major*, *L. infantum* and those of *L. tropica* belonging the zymodemes MON-307, MON-265, MON-288, MON-275, MON-54 (Figs. 2c and 4). Since the genotype HK-*Lt*G1 encompasses strains of the zymodemes MON-137 and MON-265 but the genotype PGM-G1 encompasses only strains of the zymodeme MON-137, an assay, using both underlying sequences, was employed to separate cases caused by strains of *L. tropica* of the zymodeme MON-137 from cases caused by strains of *L. tropica* of the other zymodemes mentioned. However, this assay could not be used to distinguish cases caused by strains of *L. tropica* of the zymodeme MON-307 from cases caused by strains of *L. tropica* of the other zymodemes mentioned. While this indicates a limitation to its general use compared with MLEE done using many enzyme systems, it is much simpler and less expensive to apply. Since Palestinian strains of *L. tropica* have been of either the zymodeme MON-137 or the zymodeme MON-307, one could assume that strains not shown to be of the zymodeme MON-137 are of the zymodeme MON-307. The results of this method do increase our knowledge of the genetic diversity of the species *L. tropica* and does enable the differentiation of the two sub-populations of *L. tropica* circulating in the Palestinian West Bank region.

The clustering together of Palestinian and Israeli strains of *L. tropica* of the zymodemes MON-137 and MON-265 within the genotype HK-*Lt*G1 but their separation by the genotypes of their PGM corresponds with their interrelationship based on other genetic (microsatellite and kinetoplast DNA profiles), antigenic (excreted factor (EF) serotypes), and biochemical criteria (enzyme profiles) [14], [16]. Microsatellite analysis of the Palestinian and Israeli strains of *L. tropica* belonging to the zymodemes MON-137 and MON-265 showed that they formed a single main population that separated into two subpopulations with the strains of zymodeme MON-137 falling into one subpopulation and those from the zymodeme MON-265 falling into the other; whereas the Palestinian strains belonging to the zymodeme MON-307 and the Israeli strains belonging to the zymodemes MON-288, MON-275, and MON-54 fell into another main microsatellite population (Fig. 4).

Molecular biological studies have shown that differences in the electrophoretic mobility of enzymes can be owing to heterozygosity at a single nucleotide position and are not necessarily a consequence of nucleotide diversity of the particular gene [31].

Hexokinase is not used in the Montpellier MLEE system to construct profiles based on 15 enzymes for consigning leishmanial strains to specific zymodemes and the results and separation of strains by their HK sequence presented here can only be related to and compared with zymodemes in a general manner. Figures 1 and 4 shows that the specific HK DNA sequence of the sub-group HK-*Lt*G2 corresponds with and is found in strains belonging to the zymodemes MON-58 (see [7], [8]), MON-228, MON-275 [11] and MON-307 [14] and the specific HK DNA sequence of the sub-group HK-*Lt*G1 corresponds with and is found in strains belonging to the zymodemes MON-265 [30] and MON-137 [11]. PGM is one of the 15 enzyme profiles used in constructing the 15 enzyme profiles for consigning leishmanial strains to their specific zymodemes and the results and separation of strains by their PGM sequence presented here can be related to and compared with the electrophoretic variants of PGM exposed by MLEE. Figures 2c and 4 shows that the specific PGM DNA sequence of the sub-group PGM-G2 corresponds with and is found in strains belonging to the zymodemes MON-58, MON-228, MON-275, MON-307, and in this case also MON-265, and the specific PGM DNA sequence of the sub-group PGM-G1 corresponds with and is found in strains belonging to the zymodeme MON-137. Regarding this relationship, it is interesting to note that the electrophoretic mobility of the PGM variant in the enzyme profile associated with the zymodeme MON-58 is PGM^100^ and that of the PGM variant in the enzyme profiles associated with the zymodemes MON-228, MON-275, MON-307 and MON-265 is PGM^108^ while the PGM variant in the enzyme profile associated with the zymodeme MON-137 is PGM^88^. This indicates a degree of congruity between the DNA sequence variants of PGM used here, which are based on the arrangement of their nucleotides, and the electrophoretic variants of the whole enzyme, which are based on the arrangement of their amino-acids.

The twenty-three strains of *L. tropica* from different geographical areas outside the Palestinian-Israeli region and belonging to zymodemes different from those to which Palestinian and Israeli strains belong presented PGM PCR RFLP profiles of the genotype PGM-G2 as expected. Their HK PCR RFLP profiles assigned them to the genotype HK-*Lt*G2, except for a strain of *L. tropica*, MHOM/KE/91/EB135, from Kenya that belonged to the zymodeme MON-119, a strain of *L. tropica*, MHOM/YE/86/LEM1000, from Yemen that belonged to the zymodeme MON-71 and two strains of *L. tropica*, IROS/NA/80/HD3 and MHOM/NA/84/K1 from Namibia, whose zymodemal affiliations were not known. This clustering and separation according to HK genotypes parallels that presented in a dendrogram generated by Pratlong [7], showing the interrelationship of zymodemes where the zymodemes MON-137, MON-265, MON-119 and MON-71 clustered together in their cluster (a), while other zymodemes grouped in their clusters (b) and (c).

The relatively inexpensive and simple method designed here for detecting and identifying strains of *Leishmania* in clinical samples showed that it could determine to which species, *L. major*, *L. infantum* or *L. tropica*, a local leishmanial strain belonged; and, in the case of *L. tropica*, to which zymodeme, either MON-137 or MON-307, a local strain of *L. tropica* belonged. It would be interesting to identify the sand fly vector(s) transmitting the two sub-types of the Palestinian strains of *L. tropica* described in this study to know if they are transmitted by the same vector species and, maybe, sub-species of the same vector species or each sub-type of *L. tropica* has its own vector species.

The application of the HK PCR to 61 clinical samples from patients with CL, previously confirmed by ITS1, gave 56 (91.8%) positive samples while the PGM PCR detected only 43 (70.4%) positive samples. Their consecutive RFLP patterns were highly suggestive of the zymodeme to which they belong without the need of isolating and growing the parasites to determine their enzyme profiles. However, to be absolutely sure of their zymodemal designation, their enzyme profiles should be established. Here, all the positively confirmed cases of CL were shown to be caused by strains of *L. tropica* belonging to the zymodeme MON-137. The objective here was not to compare the HK and PGM PCRs with the ITS1 PCR and confirm its results but, rather, to access the ability of the HK PCR RFLP and PGM PCR RFLP to identify the species of *Leishmania* causing a leishmaniasis and, if this was *L. tropica*, its subtype.

In summary, Two molecular biological methods based on sequences from the genes of the enzymes HK and PGM and employing PCRs and consecutive RFLPs were developed and used together to distinguish among strains of the three species *L. tropica*, *L. major* and *L. infantum* and between the two subtypes of *L. tropica* found in Palestinian foci that coincide with zymodemes MON-137 and MON-307. This could help in studying the epidemiology of the disease in Palestine.
