# Correction: Development of Assays Using Hexokinase and Phosphoglucomutase Gene Sequences That Distinguish Strains of *Leishmania tropica* from Different Zymodemes and Microsatellite Clusters and Their Application to Palestinian Foci of Cutaneous Leishmaniasis

**DOI:** 10.1371/journal.pntd.0002963

**Published:** 2014-05-28

**Authors:** The PLOS Neglected Tropical Diseases Staff

In the Acknowledgements section, the name and affiliation of Gert Van der Auwera are listed incorrectly.

The correct name is Gert Van der Auwera and he is currently at the Institute of Tropical Medicine, Antwerp, Belgium.
